# Commentary: Zebrafish as a Model for Epilepsy-Induced Cognitive Dysfunction: A Pharmacological, Biochemical and Behavioral Approach

**DOI:** 10.3389/fphar.2017.00851

**Published:** 2017-11-21

**Authors:** Avishek Amar

**Affiliations:** Department of Pharmacology, Jawaharlal Institute of Postgraduate Medical Education and Research (JIPMER), Pondicherry, India

**Keywords:** zebrafish, epilepsy, BDNF, CREB1, PTZ

This is with regards to the work “Zebrafish for studying them as a Model for Epilepsy-Induced Cognitive Dysfunction: A Pharmacological, Biochemical and Behavioral Approach” (Kundap et al., 2017). The findings of the study were shared with your esteemed journal recently. The extent of research required in case of a widely prevalent disease like epilepsy can never be enough and this is a well-known fact. In this respect, one must appreciate the author's effort to explore zebrafish as a model for epilepsy induced cognitive deficiency. Also, the idea of trying zebrafish as an animal model for this study is really heartening to see as other areas of the central nervous system too, are being explored by other researchers (Mahabir and Gerlai, 2017) but behavioral studies on zebrafish are very less in number (Kuroda et al., 2017).

But, there are certain sections in the published work which demand clarification as they raise some doubts. According to the seizure scoring scale used by the authors in their study, a lower score of 1 when the fish swims at the bottom of the tank indicates a less severe form of seizure whereas a score of 3 or 4 being a severe form of seizure when the fish swims erratically in bursts of movement. On trying to understand this claim through Figure 4 in the work by Kundap et al. we observe that the Pentylenetetrazole (PTZ) induced locomotor pattern and behavior depicts movement short swims mainly at the bottom of the tank for the group which has been administered only PTZ and no antiepileptic agent. This translates into a seizure score of 1, contrary to what the conclusion of the study indicates.

The other attempt by the authors toward a study for expression of the three genes too, demands few clarifications. For this purpose fishes were sacrificed after the behavioral studies ended which from the information provided in the study, probably is 24 h. In this regard Brain-derived neurotrophic factor (BDNF) and cAMP-responsive element-binding protein1 (CREB1) genes, deserve mention. We cannot be sure if this much time is enough to induce changes in gene expression. In one study BDNF gene expression was assessed in mice after 30 days of exercise (Sleiman et al., 2016) and in another study on rats where anti-depression treatment effects were studied on BDNF gene the expected time for expression of the same gene was taken as 2 weeks, thus giving enough time for the gene expression to change (Russo-Neustadt et al., 2000). In case of a study done on humans for assessing the levels of BDNF in depression patients on antidepressants only those patients who had received antidepressants for 3 weeks or more, were recruited in the study quite like the animal studies (Shimizu et al., 2003). Even in case of CREB1 gene, the duration required for alteration of gene expression seems to be more than 24 h as indicated by a study assessing the effects of CREB1 gene knockout in case of human breast cancer cells. Here, the cells were assessed for the same after 48 h of transfection (Zhang et al., 2013). In a study assessing kidney damage in mice by inducing a change in CREB1 expression the time interval after which the mice were sacrificed was a minimum of 4 weeks (Shan et al., 2016). In another study assessing the antidepressant effects in case of rodents again, the animals were sacrificed after 4 weeks (Li et al., 2017). The intention in all the above-mentioned studies was to allow the expression of any change induced in the genes and the effect to show clinically.

In opinion of the author of this commentary, the time point when the zebrafish were sacrificed could have been taken as 4 weeks, based on other studies done in the past or else a pilot study could have been done initially and the zebrafish could have been sacrificed at different time points, for example, post-exposure day 1, 3, 7, 14, 28 etc. and the concerned gene expression could have been studied at each time point to find out the best time interval to sacrifice the fish. This is important because it will not be appropriate to make any comments about cognitive dysfunction occurring because of change in some gene expression when we are not very sure about the induction of gene expression.

Despite the above-mentioned areas of doubt, this piece of work by Kundap et al. does help in understanding epilepsy induced cognitive dysfunction using zebrafish as a model.

## Author contributions

AA planned this general commentary and formulated the manuscript.

### Conflict of interest statement

The author declares that the research was conducted in the absence of any commercial or financial relationships that could be construed as a potential conflict of interest.
